# SFG and TG seropositivity in Humans suspected of TBD in Yucatan, Mexico

**DOI:** 10.1017/S0950268824001894

**Published:** 2025-01-08

**Authors:** Karla Rossanet Dzul Rosado, Carlos Aaron Peña Bates, Martin Raúl Tello, Henry R. Noh-Pech, Fernando I. Puerto, Oghenekaro Omodior

**Affiliations:** 1 Regional Research Center ‘Dr. Hideyo Noguchi’, Universidad Autónoma de Yucatan, Merida, Mexico; 2 Research Unit, Instituto Nacional de Enfermedades Respiratorias Ismael Cosío Villegas, Mexico City, Mexico; 3 Health Affairs Institute, West Virginia University, Charleston, WV, USA

**Keywords:** flea, Rickettsiosis, tick-borne diseases, typhus, vectors, Yucatan

## Abstract

Since 1996, the incidence of rickettsiosis has been increasing in Yucatán, Mexico, but recent prevalence data are lacking. This study aimed to determine exposure to the Spotted Fever Group (SFG) and Typhus Group (TG) in human serum samples suspected of tick-borne diseases (TBD) between 2015 and 2022. A total of 620 samples were analysed using indirect immunofluorescence assay (IFA) to detect IgG antibodies against SFG (*Rickettsia rickettsii*) and TG (*Rickettsia typhi*), considering a titer of ≥64 as positive. Results showed that 103 samples (17%) were positive for *R. rickettsii* and 145 (24%) for *R. typhi*, while 256 (41%) and 229 (37%) were negative, respectively. There was a cross-reaction in 244 samples (39%). Individuals with contact with vectors, such as ticks, showed significant exposure to fleas (p = 0.0010). The study suggests a high prevalence of rickettsiosis and recommends prospective studies to assess the disease burden and strengthen surveillance and prevention in Yucatán, considering factors like temperature and ecological changes.

## Key results

- The study suggests a high prevalence of TB-Rickettsiosis in Yucatan, Mexico.
- The 25 to 54 years age group had the highest prevalence of rickettsial IgG antibodies.
- The presence of rickettsial IgG antibodies in the age group 0 to 5 years, suggests early exposure to disease vectors in Yucatan, Mexico.

## Introduction

Tick-borne diseases (TBD) have increased dramatically in recent years. This adjustment is associated with climatic changes due to global warming and socio-cultural factors that favour greater contact with disease-carrying vectors [1–3].

Rickettsiosis is caused by strict intracellular bacteria of the genus *Rickettsia* and transmitted by fleas, lice and ticks. *Rickettsias* have been classified into four major groups according to their antigenic and clinical properties: the ancestral group (AG), the transitional group (TRG), the spotted fever group (SFG), and the typhus group (TG) [4]. The SFG causes the most severe and symptomatic disease of the genus. Cases of Rocky Mountain Spotted Fever (RMSF) caused by *Rickettsia rickettsii* are the most severe reported in the Americas. [5] Other reported cases are of Murine Typhus, caused by *Rickettsia typhi*, for which the specific route of infection is not yet known. Cases have been reported worldwide, mainly in South America, Mexico, the Middle East, Africa, Asia, and Australia [6].

At least one case of rickettsiosis has been reported in each of Mexico’s states, with the highest number of cases recorded in areas with warm and humid climates that allow the pathogen to develop more fully [7]. By 2020, 14 species of *Rickettsia* associated with 26 species of arthropods and 17 species of mammals will have been recorded in Mexico [8]. Yucatan, Mexico is considered an endemic area for many vectors, mainly associated with environmental and ecological characteristics that favour the proliferation of the life cycle of *Rickettsias.* The presence of at least four *Rickettsia* species in symptomatic patients has been implicated in this region: *Rickettsia felis*, *R. typhi, Rickettsia parkeri*, and *R. rickettsii.* The latter particularly in paediatric patients who have presented with a resolution of the disease under treatment with chloramphenicol and oral endovenous doxycycline [9, 10].

Since the 2000s, there have been numerous molecular studies for diagnosis in humans and identification in animals and ticks, but there are no recent data on the seroprevalence of SFG and TG species in the population. Indirect immunofluorescence assays (IFA) serological tests are a fundamental tool in identifying *Rickettsia* and offer notable advantages over molecular methods for epidemiological studies. These tests, which are used to detect antibodies in response to infections, provide a valuable means of understanding the epidemiology of *Rickettsia* infections, which is crucial for implementing public health strategies in endemic or high-risk regions [11, 12].

Furthermore, 28 years after the first serological study, there is no current data on exposure to TB-Rickettsiosis, especially given the ecological changes in the area. The current study aims to determine the exposure to Spotted Fever Group (SFG) and Typhus Group (TG) in subjects with a history of contact with ticks and fleas during 2015–2022 in Yucatan, Mexico.

## Materials and methods

### Study design, population, and sample collection

A descriptive study was conducted on 620 human samples collected between 2015 and 2022, Given a historical record of exposure to fleas and ticks. Blood samples were obtained using tubes containing a clot activator and serum separator gel (BD Vacutainer 367,863 and 368,159, respectively; Franklin Lakes, NJ, USA). The samples were then centrifuged at 3,500 rpm for 10 min at room temperature to obtain the serum, which was subsequently transferred and stored in a cold environment. Aliquots of serum were prepared in volumes of 300 to 500 μL and transferred to 1.5 mL microtubes for storage at −20 °C until required for analysis. For molecular diagnosis, a whole blood sample was collected in 3.8% sodium citrate as an anticoagulant, and DNA was immediately extracted using a QIAamp DNA kit (QIAGEN, Valencia, CA, USA), following the manufacturer’s instructions. Molecular identification of Rickettsia was performed by 17 kDa single-step PCR and nested PCR, which allowed differentiation between Rickettsia SFG and TG by amplification of ompB gene fragments [13, 14]. Providers completed the health form at the time of initial contact and collected data, including (a) demographics (gender, age, origin), (b) history of tick bite and/or direct contact with dogs, (c) presence of vectors in the region, (d) confirmed cases in the locality and (e) history of visit or residence in areas with rickettsiosis transmission were considered.

### Serology tests

Indirect immunofluorescence assay (IFA) was carried out using autochthonous antigens of *R. rickettsii* and *R. typhi* (representative of the spotted fever group and typhus group *Rickettsiae*).

*R. rickettsii* and *R. typhi* were cultured in Vero cells, and after an infection rate ≥ 70% the cells were harvested and deposited in antigen prepared according to standard procedures. In-house indirect fluorescent antibody assays were performed on the samples to detect antibodies reactive. Dilutions were prepared in a series of human serum in phosphate-buffered saline (PBS) containing 1% and 20% bovine serum albumin (BSA). Antigen plaques were blocked in PBS containing 1% BSA and 0.01% sodium azide; 10 μL of each serum dilution were added to each well of the antigen sheet and incubated in a humid chamber for 30 min at 37 °C. The sheets were then washed with PBS containing Tween 20% to 0.1% for 10 min and then washed twice in the same solution for 10 min. Fluorescein isothiocyanate–conjugated goat anti-human IgG e IgM immune serum (Kirkegaard and Perry Laboratories, Gaithersburg, MD) diluted 1:100 in PBS containing 1% BSA and 0.01% Tween20 was added to each well and incubated in a humid chamber for 30 min at 37 °C. Slides were washed once with PBS containing 0.1% Tween 20 for 10 min and once with PBS containing 0.1% Tween20 and 0.01% Evans blue for 10 min and observed under a fluorescence microscope at 400X magnification. Serum samples yielding distinctly fluorescent *Rickettsiae* sp. at a ≥ 64 dilution were considered positive. [15–17]

### Ethical considerations

The Research Ethics Committee of the O’Horan Hospital (Merida, Yucatan, Mexico) approved the ethical statements accompanying this study, as a goal of project CIE-010-1-14. The authors assert that all procedures contributing to this work comply with the ethical standards of the relevant national and institutional committees on human experimentation and with the Helsinki Declaration of 1975, as revised in 2008.

### Statistical analysis

Collected data were analysed using GraphPad 9.0, Software, Inc. To analyse differences between groups of interest, statistical significance for dichotomous variables was assessed using the Chi-square χ^2^ test. The Shapiro–Wilk normality test was used to evaluate the data distribution, indicating that our data did not follow a normal distribution. Statistical analysis was performed using the Kruskal-Wallis test and corrected by Dunns’ test to compare between groups. P values <0.05 were considered statistically significant.

## Results

From 2015 to 2022, 620 human serum samples were analysed. The samples included in the study were from municipalities of Yucatan: Merida, Progreso, Kantunil, Dzita, Ucu, Izamal, Sotuta, Tahmek, Oxkutzcab, Kanasin, Hocaba, San Felipe, Timucuy, Uman, and Cenotillo. Demographic data showed that 58% (359/620) were female and 42% (261/620) were male. The mean age of the patients was 27 years (range: 1 month to 79 years). In terms of exposure, contact with ticks was the most common, followed by contact with ticks and fleas, and finally fleas alone. However, a significant proportion refused to disclose whether they had been exposed to any vectors or claimed to be unaware of such exposure (Table 1).

**Table 1.** Demographic characteristics of the subjects

Demographic characteristics n (%)	
**Gender**	
Male	261 (42)
Female	359 (58)
**Age in years m.d.:36 (5.8)**	
de 0 a 5	74 (11.9)
de 6 a 15	157 (25.3)
de 16 a 24	96 (15.5)
de 25 a 54	208 (33.6)
> 55	49 (7.9)
**Vector exposure**	
Ticks	186 (30)
Fleas	38 (6.1)
Ticks and fleas	147 (23.7)
Other vectors	106 (17.1)
Unknow	143 (23.1)
**Samples included per year**	
2015	78 (12.6)
2016	99 (16)
2017	107 (17.2)
2018	108 (17.4)
2019	132 (21.3)
2020	52 (8.4)
2021	21 (3.4)
2022	23 (3.7)

m.d.* = missing data.

**Figure 1.**
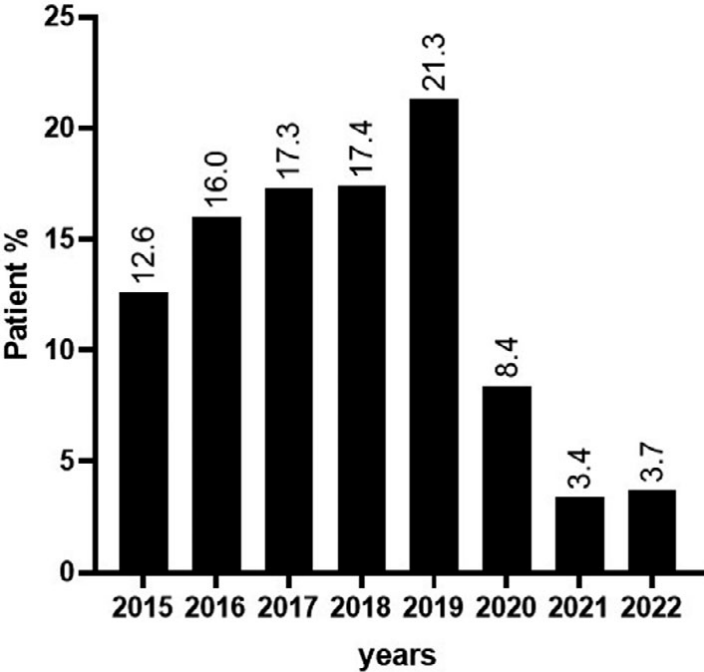
Distribution expressed as a percentage of the subjects included in the study for each year.

The distribution of subjects in this study gradually increased over the years, which is an expected trend. However, the number of human serum samples tested was drastically affected at the beginning of the COVID-19 pandemic, with the number of individuals with symptoms sent for IFA testing decreasing from 132/620 (21%) to 21/620 (3%) (Figure 1).

Out of the 620 human serum samples included in this study, of these individuals 103 (17%) were positive with titer ≥64 which is the established cut-off point for TB-Rickettsiosis endemic areas and 256 (41%) were negative for *R. rickettsii.* On the other hand, 145 (24%) were positive with titer ≥64 and 229 (37%) were negative for *R. typhi.* Regarding the cross-reaction, 244 (39%) had the same result for both species (Table 2).

**Table 2.** Distribution of serum samples according to positivity with respect to antibody titer obtained

IgG titer	*Rickettsia rickettsii*			*Rickettsia typhi*			Cross reaction
	Total	Negative	Positive	Total	Negative	Positive	Total
< 64	227	227	0	189	189	0	–
≥ 64	72	18	33	105	32	52	21
128	54	11	34	32	8	15	9
256	20	2	14	14	0	10	4
512	153	0	4	184	2	33	149
1,024	94	0	32	96	0	35	61

The 512 titer has the highest proportion of cross-reacting samples, 149 (24%), followed by the 1,024 titer (10%), suggesting a high percentage of contact with *Rickettsia* sp. and subjects of recent illness. Of the group of individuals negative for antibodies to both species, the results can be taken with caution given that they present clinical symptoms suggestive of rickettsiosis, so it could be thought of as another species or another pathogen (Table 2).

When analysing the Odds Ratio (OR) for antibody titers ≥1:64 in relation to gender, significant differences were observed [P = 0.0035 OR (95% CI) = 0.4639 (0.2809 to 0.7681)] with similar P values in both groups evaluated, suggesting that women in this population group studied have a higher odd of becoming infected by *Rickettsia* sp. than men (Table 3).

**Table 3.** SFG and TG-IgG seropositivity

IgG titer	*Rickettsia rickettsii* (n)				*Rickettsia typhi* (n)				Cross reaction	
	P		N		P		N		P	
n = 620	M	F	M	F	M	F	M	F	M	F
< 64	0	0	55	59	0	0	21	54	0	0
≥ 64	10	23	5	13	25	27	10	22	6	15
128	9	25	7	4	6	9	2	6	5	4
256	3	11	1	1	8	2	0	0	0	4
512	2	2	0	0	13	20	1	1	70	79
1,024	10	22	0	0	17	18	0	0	23	38
	P = **0.0035** OR (95% CI) = 0.4639 (0.2809 to 0.7681)				P = **0.0035** OR (95% CI) = 0.4639 (0.4416 to 0.8559)					

χ^2^ comparison of seropositive status (endpoint titers ≥64) by sex; P: Positive, N: Negative, M: male, F: female.

**Figure 2.**
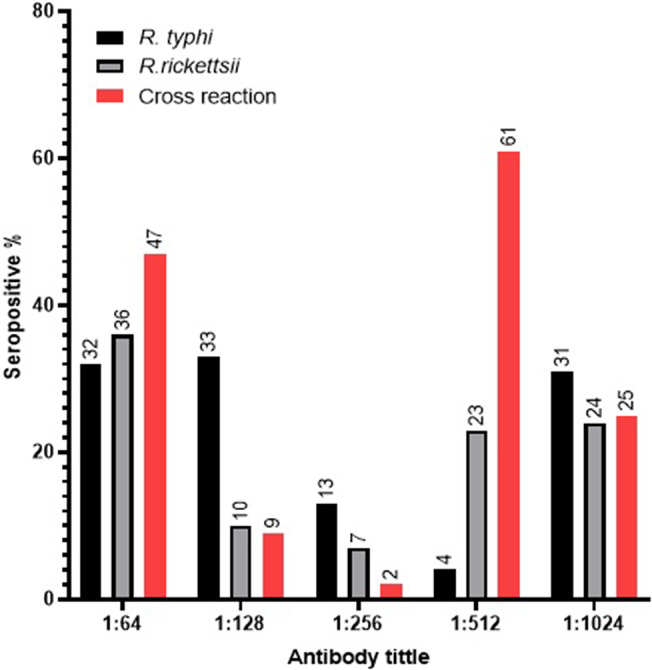
Representation of antibodies and positive human serum samples according to titer with respect to rickettsia species. The graph represents the proportion of seropositive patients in the titers evaluated in the *Rickettsia typhi*, *Rickettsia rickettsii* and cross-reaction slides.

When comparing the antibody response considering the positive slides, a higher percentage of serum samples with high antibody titers can be observed, both in each of the species and in the cross-reaction. Evidencing the high prevalence of IgG antibodies in the studied population (Figure 2).

**Figure 3.**
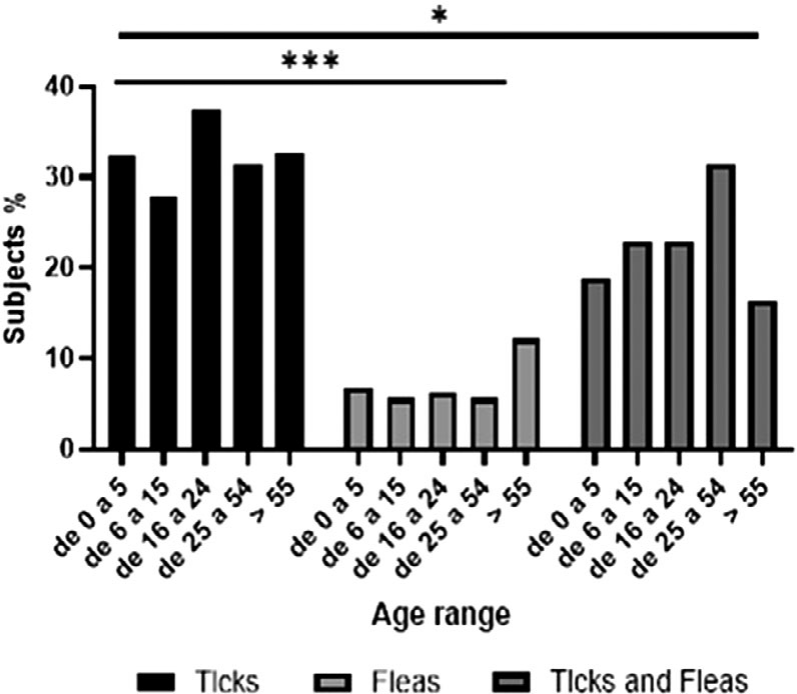
Representation of the percentage of human serum samples according to the age ranges evaluated to determine their exposure to the different vectors. The differences in all age ranges between ticks and fleas are significant; on the other hand, there is a significant value when comparing the groups exposed to ticks and the fleas and ticks set. Statistical analysis was performed using the Kruskal-Wallis test and corrected by the Dunns test to compare between groups. ***p < 0.001 and *p < 0.05.

A comparison was made between the different age groups and it was observed that the perception of exposure to ticks is significantly higher than that of fleas. (p = 0.0010). However, significant differences were also observed in subjects with exposure to both fleas and ticks (p = 0.034) (Figure 3). The patients in the study had higher titers to *R. typhi* than to *R. rickettsii*, which may suggest a change in the mode of transmission. This could indicate that ticks, rather than fleas, are the primary vector in the population being evaluated. Therefore, further molecular studies are needed.

## Discussion

The initial reports of Rocky Mountain spotted fever in northern Mexico date back to 1940. In 1993, a study identified a group of patients with febrile characteristics and no presence of dengue antibodies in samples from Yucatan and Jalisco. Upon analysing this group of samples using the IFA technique, IgG antibody reactivity was obtained in 40% of the sera, some of which belonged to the state of Yucatan. This marked the beginning of the first reports of rickettsia in southeastern Mexico [18].

Although direct immunofluorescence is a standard method for serological studies and is valuable, it has limitations. For example, cross-reactivity between *Rickettsia* species makes it difficult to accurately differentiate species without additional studies. Furthermore, the interpretation of results is subjective, which can affect the consistency and comparability of data. Trained personnel are required for this method [11].

This study analysed antibody testing results using the IFA technique from 2015 to 2022. The findings indicate that 103 (17%) were positive with titer ≥64, which is the established cut-off point for TB-Rickettsiosis endemic areas, and 256 (41%) were negative for *R. rickettsii*; on the other hand, 145 (24%) were positive with titer ≥64 and 229 (37%) were negative for *R. typhi.* Regarding the cross-reaction, 244 (39%) had the same result for both species.

The high cross-reactivity and high prevalence of antibodies at titers ≥64 suggest the potential for increasing the cut-off point in the Yucatan population, which is highly exposed to rickettsial infections. This could result in modifications to the data established in different regions of the world. Although this threshold may not indicate active infection in all cases, it provides a valuable measure of population exposure and can be reviewed and adjusted based on local and epidemiological data.

This study shows a joint positivity of the two representative species of 248 (40%). however, considering the cross-reaction, a total of (492) 79% of positive samples were collected in a group of individuals with symptoms suggestive of rickettsiosis. Suggesting a high exposure to SFG and TG in the state of Yucatan. When comparing with previous reports in Yucatán, it is evident that there is a gap in exposure to rickettsial infections. In 1999, Zavala et al. [18] reported 5% of *Rickettsia akari* and 0.8% of sera positive for IgG antibodies against *R. rickettsii/R. typhi.* A study conducted by Peniche Lara et al. [16] in Yucatan found that the prevalence of *R. typhi* in vectors of marginalized populations was 1.8% (6/354). It is important to note that Zavala recruited volunteers from 60 municipalities of Yucatan, whereas our study is based on samples from patients with clinical symptoms of a febrile illness but negative for other diseases such as dengue, chikungunya and Zika. Therefore, as this is a classically directed analysis, we cannot extrapolate the high number of positive cases to statistical data from the general population of Yucatan.

The cross-reactivity observed in the species tested in this study is higher than the 20% reported for *R. typhi* and other rickettsial species [19]. This may result in misclassifying the patient’s diagnosis, underscoring the necessity for a second blood sample to be obtained 15 to 60 days after the initial sample and a comprehensive clinical description of the patient. Furthermore, it underscores the necessity of meticulously establishing novel antibody titers in endemic regions to ascertain the precise antibody level. It could then be suggested that the need to include a panel of antigens from different *Rickettsia* species could help to better differentiate specific immune responses and reduce confusion due to cross-reactivity. This would be useful in clinical and epidemiological contexts, although it would require investment in more complex assays. Continuous monitoring and updating of surveillance strategies in endemic areas of different species and adaptation of surveillance and control strategies according to updated data, always considering the limitations of serology and the impact of cross-reactivity.

The prevalence of antibodies in population groups is crucial for epidemiology and developing effective strategies to prevent TB-Rickettsiosis. Environmental changes linked to global warming have led to an increase in disease-transmitting vectors and greater contact with accidental hosts, including humans. [3] Social and cultural changes have contributed to the increase of domestic and peri-domestic animals, which in some cases may be infested by ectoparasites [15]. This study found that the circulation of *R. typhi* and *R. rickettsii* is active and high, as previously reported in the southeast [16, 17, 20, 21]. However, there is still no data on the specific time at which the spread of TB-Rickettsiosis began in rural and urban communities in Mexico and Latin America [22].

A notable finding is the presence of IgG antibodies in patients aged 0 to 5 years, including infants, with antibody titers that can be explained by contact with fleas (mainly *Ctenocephalides felis*, cat flea, and *Xenopsylla cheopis*, or mouse flea) or ticks (mainly *Rhipicephalus sanguineus*, *Rhipicephalus microplus*, and *Amblyomma mixtum*) in the home environment where they spend most of their time. This was demonstrated by Dzul Rosado et al. in a study with children from rural populations [16, 23]. Identifying antibodies in young children indicates early exposure and potential transmission foci in the domestic environment. This is a concerning finding, given that infection by *R. rickettsii* in children is relatively common but can, in many cases, result in fatality [24]. Understanding the prevalence of this infection is crucial for developing more targeted health campaigns.

The age group with the highest prevalence of IgG antibodies is the 25 to 54-year-old group, which historically comprises the majority of the working population. It should be noted that this is a subjective evaluation. Although the type of economic activity is unknown, it is well documented that in most municipalities of the state of Yucatan, individuals engage in outdoor work. This work involves contact with rodents, farm animals, and/or agriculture, which requires working in deforested areas or fragmented forests, increasing the risk of contracting vector-borne infections. Opossums are a common factor in the amplification of TB-Rickettsiosis [25] .

The seropositive IgG results in this population suggest past exposure to *Rickettsia*, which is relevant for epidemiologic studies and for understanding seroprevalence in vector-exposed populations. Importantly, a negative PCR result was determined for each sample and added to the specificity of the IFA, providing a clearer picture of the absence of current infection and reducing the uncertainty associated with serologic cross-reactivity, providing a more robust basis for public health decision-making.

This study reports a higher proportion of women than men, which contradicts findings from previous studies [26–28]. The higher proportion of women may be due to their continued involvement in domestic and agricultural work in our state [P = 0.0035 OR (95% CI) = 0.4639 (0.2809 to 0.7681)]. An alternative explanation is that the observed difference is the result of randomization concerning gender in the sample population or bias in the lack of attendance of men at medical centres. It has been observed that the female population attends health centres more frequently, possibly due to gender issues in the state and the ingrained customs in question that women are responsible for cleaning, caring for backyard animals, and performing many activities in areas of high vector presence [29].

The study shows a higher TG-seropositivity of antibodies reactive to *R. typhi* than to *R. rickettsii.* A recent study conducted in Bolmay, Yucatan reported *R. typhi* infections in *M. musculus* and *Rattus rattus*, which are the most abundant rodent species in domestic and peridomestic environments in the state and serve as reservoirs. The study suggests a wide distribution of *R. typhi* in the region, based on a previous report that found a 25% frequency of *R. typhi* infection in animals captured in Merida, including *R. rattus.*

It is important to investigate whether recent real estate developments around the capital city have influenced the results obtained here. As the risk of infectious diseases such as murine typhus is mainly associated with the urban environment, in the same way, the flea sticks and then it is not easy to find it because of its way of life, compared to the tick that bites and roots itself on the skin [30, 31].

The tested sera show clear cross-reactions between different *Rickettsia* species and even with other bacterial genera. These reactions are usually triggered during murine and epidemic typhus infections, as well as SFG infections, although less frequently. [32] It is not possible to consider it a coinfection since there is very little evidence of coinfections by *R. typhi* and *R. rickettsii* in humans. A study carried out in South Korea reported that only 3.5% of the samples seropositive for SFG rickettsiae were also seropositive for TG rickettsiae. The species found were *Rickettsia conorii* and *R. typhi*, which are the main circulating species in that region [14].

IFA is a widely used methodology for epidemiological evaluations because high antibody titers are not expected in the first days of infection. Depending on the titers obtained in the IFA, the responsibility of the infection can be attributed to the species that causes more reactivity. However, it is important to note that this technique may not be as specific as culture or molecular methods. Additionally, a single IgG result does not necessarily indicate an active rickettsial infection but rather may indicate a past infection at an undetermined time [27]. Therefore, the correct interpretation of serological results of rickettsial infection must take into account the clinical history of the patient and the baseline reactivity data of the population (especially in endemic areas), since currently, the positive antibody response may be due to non-specificity and different surface antigens of the rickettsial species, considering that in Yucatan the reactivity may be due to *R. typhi, R. rickettsii*, *R. akari, Rickettsia felis* and *R. parkeri* [33]. To increase the technique’s specificity, it is recommended to use different antigens given the diversity of *Rickettsia* species circulating in Yucatan.

It is important to note the impact of the COVID-19 pandemic on the diagnosis of other infectious and non-communicable diseases. This study observed a drastic decrease in the diagnosis of probable cases of *rickettsiae* during the SARS-CoV-2 pandemic. As a result, this disease was neglected, and it is difficult to predict how many cases had a fatal outcome due to a lack of timely diagnosis or correct treatment. Due to the disruption of medical services and the saturation of systems, there has been an exclusive focus on patients with COVID-19 disease. This has resulted in a significant impact on non-COVID-19 patients who require medical attention. It is important to address this issue and ensure all patients receive the necessary care [34].

It is imperative to prioritize prospective studies and develop concrete public health intervention strategies to address rickettsial infections in Yucatan. It is recommended that longitudinal and ecological studies continue to be conducted to gain a deeper understanding of the local epidemiology, prevalence of infections, and region-specific vectors and reservoirs, as reported in the entity [35]. The results of these investigations will assist in identifying areas and periods of elevated risk for transmission, thereby providing a robust foundation for implementing effective control measures.

## Conclusion

This study presents 70% SFG and TG IgG seropositivity in samples with suspected TBD in Yucatan, 28 years after the first serologic report of TB Rickettsiosis.

A comprehensive examination of the social characteristics of the Yucatecan population is essential for an accurate assessment of the primary risk factors associated with this disease. This knowledge is vital for the development of effective preventive strategies that can be implemented in various ways to reduce the prevalence of the disease:

(1) Implementing active surveillance systems that include monitoring networks in rural communities and urban centres, complemented by public education campaigns to raise public awareness about the prevention and control of these vectors, is recommended. These programs should place an emphasis on personal protection measures and the promotion of hygiene and environmental management practices that serve to reduce exposure to ticks and other vectors.

(2) Improved diagnostic capabilities and ongoing training of health professionals are needed to ensure the rapid identification of rickettsial infections.

Implementing these recommendations will reinforce the regional health response and contribute to mitigating the spread of rickettsial-borne diseases in Yucatan.

## Data Availability

The data that support the findings of this study are available from the corresponding author upon reasonable request.
